# Non-Invasive Characterization of Lower Extremity Deep Vein Thrombosis via Advanced Ultrasound Elastography: A Prospective Pilot Study

**DOI:** 10.1007/s11739-025-04135-7

**Published:** 2025-10-07

**Authors:** Nicola Potere, Mattia Giulianelli, Matteo Candeloro, Maria Domenica Guglielmi, Silvana Pardi, Marcello Di Nisio, Ettore Porreca

**Affiliations:** 1 Department of Medicine and Ageing Sciences, “G. d’Annunzio” University of Chieti–Pescara, Chieti, Italy; 2 Department of Medicine, University of Ottawa at The Ottawa Hospital and the Ottawa Hospital Research Institute, Ottawa, ON Canada; 3 Division of Internal Medicine, Department of Medicine, “F. Veneziale” Hospital, Isernia, Italy; 4 Department of Innovative Technologies in Medicine and Dentistry, “G. d’Annunzio” University of Chieti–Pescara, Chieti, Italy; 5 Vascular Medicine and Thrombosis Clinic, Division of General Internal Medicine, Department of Medicine, “SS. Annunziata” Hospital, Chieti, Italy

**Keywords:** Ultrasonography, Clot, Elasticity imaging, Thrombus elasticity, Vascular imaging, Venous thromboembolism

## Abstract

Duplex ultrasonography is the preferred non-invasive imaging technique for diagnosing lower extremity deep vein thrombosis (DVT), but considered largely unreliable for determining thrombus-specific features. Elastosonography techniques including real-time strain elastography (RT-SE) are widely used in clinical practice to assess tissue elasticity, but its potential remains largely unrealized in DVT, where RT-SE might provide adjunct information on thrombus biomechanical properties potentially aiding clinical decision-making. This single-center prospective pilot study investigates the use of modern RT-SE imaging enhanced with an advanced quantification software for reconstructive tissue elasticity analysis in patients with symptomatic acute lower-extremity DVT objectively diagnosed within 6 months of enrollment. Between-group differences in median thrombus elasticity, assessed via a newly proposed elasticity biomarker, namely thrombus tissue deformation index, were evaluated using Mann–Whitney U or Kruskal–Wallis tests. Relationships between thrombus elasticity and patient-, DVT- and anticoagulation-related factors were assessed through Spearman’s rank-order correlation. Thirty-eight deep venous thrombi were analyzed using advanced RT-SE, in addition to standard duplex ultrasonography (i.e., triplex ultrasonography). Median thrombus elasticity was 25.7% (interquartile range [IQR], 22.1–26.1), 20.8% (IQR, 17.1–22.1), and 3.8% (IQR, 1.3–6.6) in acute, sub-acute, and chronic DVT, respectively (*p* < 0.001). Thrombus elasticity robustly correlated with the period of DVT diagnosis (i.e., lower elasticity with increasing thrombus chronological age; *ρ* = −0.70, *p* < 0.0001). Elasticity of chronic thrombi inversely correlated with dynamic changes in residual vein obstruction (i.e., higher elasticity of thrombi undergoing smaller size reduction over time; *ρ* = 0.85, *p* < 0.0001). These findings preliminarily identify modern advanced RT-SE imaging as a viable and easily-implementable tool for enhanced non-invasive thrombus characterization, with the potential to inform thrombus-guided management strategies in patients with DVT.

**Graphical Abstract:**

Framework for thrombus elasticity assessment via advanced real-time strain elastography, and potential clinical usefulness of enhanced non-invasive DVT characterization.

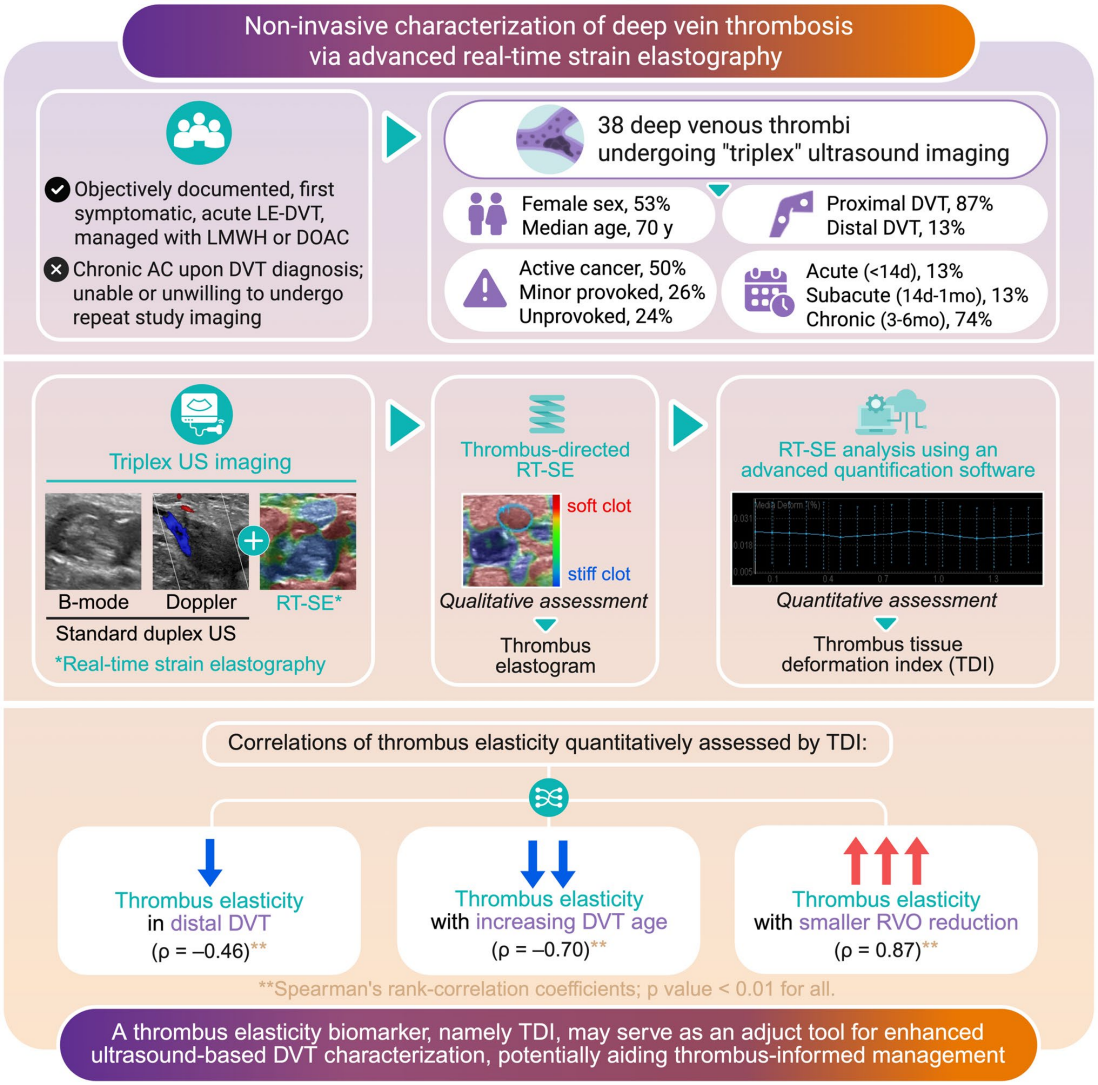

## Introduction

Duplex ultrasonography is the preferred non-invasive imaging technique for the diagnosis of lower extremity deep vein thrombosis (DVT), but it is considered largely unreliable in determining thrombus-specific features [1, 2]. Enhanced ultrasound-based DVT characterization would provide easily accessible, readily available thrombus-specific information potentially aiding diagnostic and therapeutic management [3]. Shear-wave and real-time strain elastography (RT-SE) are widely used in clinical practice to assess elasticity of diverse tissues including liver, breast, and myocardium [4], but their potential remains largely unrealized in vascular disease [5]. It has been proposed that RT-SE might help dating DVT based on thrombus biomechanical properties [3]. This might be particularly relevant in informing therapeutic decisions across frequent challenging scenarios, including in: (i) patients with suspected recurrence, in whom new-onset/worsening signs and symptoms could be attributable to a newly-formed clot or to the post-thrombotic syndrome; and (ii) asymptomatic patients with history of DVT, in whom it might be unclear whether the presently observed thrombus represents a fresh new clot or the old thrombus residue [6]. Older studies suggested promise for RT-SE in discriminating acute from non-acute DVT, but these employed qualitative or pseudo-quantitative approaches for thrombus elasticity estimation, which are associated with inherent substantial methodological limitations [7]. Technological advances have led to the development and clinical implementation of advanced RT-SE-based analysis software enabling more accurate and standardizable quantitative tissue elasticity assessment [4, 8]. Leveraging an advanced quantification software for RT-SE-based reconstructive imaging analysis, we evaluated the use of thrombus-directed tissue deformation index (TDI) as an ultrasound-based thrombus elasticity biomarker in patients with lower extremity DVT, and explored associations of thrombus elasticity with patient-, DVT- and treatment-related factors.

## Methods

Ambulatory patients aged > 18 years diagnosed (≤ 6 months) by compression ultrasonography (CUS) with first symptomatic acute lower extremity DVT were eligible for participation in this prospective single-center observational pilot study. Patients were considered ineligible if unwilling/unable to comply with study protocol/procedures, or with conditions (e.g., agitation, tachypnea, arrhythmia) potentially interfering with ultrasound imaging execution/interpretation, or on chronic anticoagulation at the time of DVT diagnosis. The study was conducted at the Vascular Medicine and Thrombosis Clinic, “SS. Annunziata” Hospital, Chieti, Italy, approved by the local institutional review board, and conducted in accordance with the Declaration of Helsinki’s principles for medical research involving human subjects. Upon enrollment, a study investigator collected patient’s clinical data, including demographics, DVT diagnosis date, risk and provoking factors, and anticoagulant therapy (administered at the discretion of treating physicians). Subsequently, participants underwent repeat study imaging consisting in triplex ultrasonography, comprising advanced RT-SE in addition to duplex ultrasonography [3]. Imaging was performed by the same experienced sonographer blinded to the patient’s clinical data, according to a predefined standardized protocol based on currently recommended criteria [1, 2, 9, 10]. Briefly, proximal and distal deep veins were systematically scanned, and assessed for compressibility on the transverse plane [1, 2, 10]. Residual vein obstruction (RVO) was determined by measuring thrombus size in anteroposterior diameter [1, 2]. If participants had multiple thrombi simultaneously detected in clearly distinct veins upon index DVT diagnosis, data regarding each thrombus were recorded. Imaging was performed using Affiniti 30G Ultrasound System with a high-resolution linear 5–12 MHz transducer (Philips Ultrasound, Bothell, WA, USA).

RT-SE assesses tissue biomechanical properties in response to deformation using quasi-static methods based on Young’s elasticity modulus, accounting for compressive stress in relation to strain or proportional deformation [4, 9]. Tissue deformation can be induced artificially by applying repeated micro-pressures through the transducer, or by exploiting internal physiological cardiorespiratory motion holding the transducer steady [4, 9]. Since the former method carries greater intrinsic operator dependency and variability, the latter was adopted [4, 9]. Similar to the CUS procedure, the pression exerted during RT-SE execution coincided with the pressure exerted to achieve thrombus compression, which was simultaneously monitored in B-mode to ensure constant intensity, homogenous compression [2, 9]. After launching the RT-SE module, a real-time color-coded elastogram (i.e., qualitative output) was generated using a fixed elastogram box size [4, 9, 11]. The elastogram displayed relative tissue elasticity, conventionally indicating elastic/soft areas in blue, intermediate-elastic areas in green, and inelastic/stiff areas in red [4, 9, 11]. A region of interest was thrombus-centered, and manually optimized to precisely match with thrombus borders. For each thrombus examined, a dynamic 3-s color-coded elastogram recording was acquired and exported in digital imaging and communications in medicine (DICOM) format for quantitative analysis using Philips QLab 15.0 advanced quantification software and Elasticity Quantification Q-App (Philips Ultrasound, Bothell, WA, USA) [11]. The tissue deformation function embedded in the software was used for quantitative thrombus elasticity estimation. For each thrombus elastogram, a normalized mean thrombus TDI value (i.e., quantitative output) was computed by the software using parametric image and motion compensation methods [11]. Higher TDI values indicated higher deformation/elasticity (“softer” thrombi), whereas lower TDI values denoted lower deformation/elasticity (“stiffer” thrombi).

Participants were grouped based on the time from CUS-confirmed DVT diagnosis to repeat study imaging. Participants in whom diagnosis was made < 14 days were included in the acute DVT group, whereas those with an older diagnosis in the non-acute DVT group. These were further categorized in sub-acute (14 days–1 month) and chronic DVT (3–6 months). For chronic DVTs, thrombus size measured at the time of DVT diagnosis was retrieved from the baseline ultrasound report, and compared with that measured upon repeat study imaging. Delta RVO (ΔRVO) was defined as percentage change in thrombus size between DVT diagnosis and repeat study imaging.

Continuous and categorical variables were expressed as median and interquartile ranges (IQRs), and counts and percentages, respectively. Between-group differences in median thrombus TDI values were assessed using Mann–Whitney U test or Kruskal–Wallis test, as appropriate. Spearman’s rank-correlation coefficients (ρ [rho]) were calculated to evaluate factors correlating with thrombus elasticity, and correlation matrices generated. Univariable linear regression was used to model the association between log-transformed thrombus TDI (dependent variable) and ΔRVO. A two-tailed *p* value < 0.01 was set to indicate statistical significance. Analyses were performed using R version 4.2.3 and Rstudio version 2023.9.0.463 for macOS.

## Results

Out of 376 subjects undergoing duplex ultrasonography for suspected DVT at our center between July 1 st and October 31 st 2023, 48 were objectively diagnosed by CUS with first symptomatic acute lower extremity DVT. Of these, nine were ineligible, six unable to return to the center, and three did not provide consent for participation. Thirty patients (53.3% females; median age, 70 years) were ultimately included in the study, leading to 38 deep venous thrombi available for analysis (Table 1). Of these, 29 (76.3%) were considered to be provoked (50.0% by cancer, 26.3% by transient provoking factors), and 9 (23.7%) unprovoked. Thirty-three (86.8%) thrombi were proximal, and five (13.2%) distal. Eighteen (47.4%) were managed with low-molecular-weight heparin (LMWH) followed by a factor Xa inhibitor, fourteen (36.8%) with factor Xa inhibitor alone, and six (15.8%) with LMWH alone. Anticoagulation was administered at therapeutic doses starting from DVT diagnosis. All patients were on treatment at the time of the study visit.

**Table 1:** Characteristics of the study participants and the deep venous thrombi examined

	*n* (%)
Patient age (median [years], IQR)	70.0 (58.3–72.8)
Female sex	16 (53.3)
Nature of DVT^a^	
Provoked	29 (76.3)
Active cancer	19 (50.0)
Transient provoking factor	10 (26.3)
Unprovoked	9 (23.7)
Location of DVT	
Proximal DVT	33 (86.8)
Common femoral vein	8 (21.1)
Femoral vein	10 (26.3)
Popliteal vein	15 (39.5)
Distal DVT	5 (13.2)
Time since DVT diagnosis^b^	
Acute DVT (< 14 d)	5 (13.2)
Non-acute DVT (14 d – 6 mo)	33 (86.8)
Subacute DVT (14 d – 1 mo)	5 (13.2)
Chronic DVT (3 mo – 6 mo)	28 (73.7)
Anticoagulant therapy for DVT	
LMWH followed by factor Xa inhibitor	18 (47.4)
Factor Xa inhibitor alone	14 (36.8)
LMWH alone	6 (15.8)

Values refer to absolute numbers and percentages, if not otherwise specified; percentages may not add up to 100 due to rounding. Age and sex refer to the overall number of enrolled patients (*n* = 30); DVT characteristics refer to the overall number of deep venous thrombi examined (*n* = 38)

*d* days, *DVT* Deep vein thrombosis, *Factor Xa* Activated factor X, *IQR* Interquartile range, *LMWH* Low-molecular-weight heparin, *mo* months

^**a**^DVT provoking factors during 3 months preceding DVT diagnosis

^**b**^Defined as the time from CUS-confirmed DVT diagnosis to repeat study imaging

**Fig. 1:**
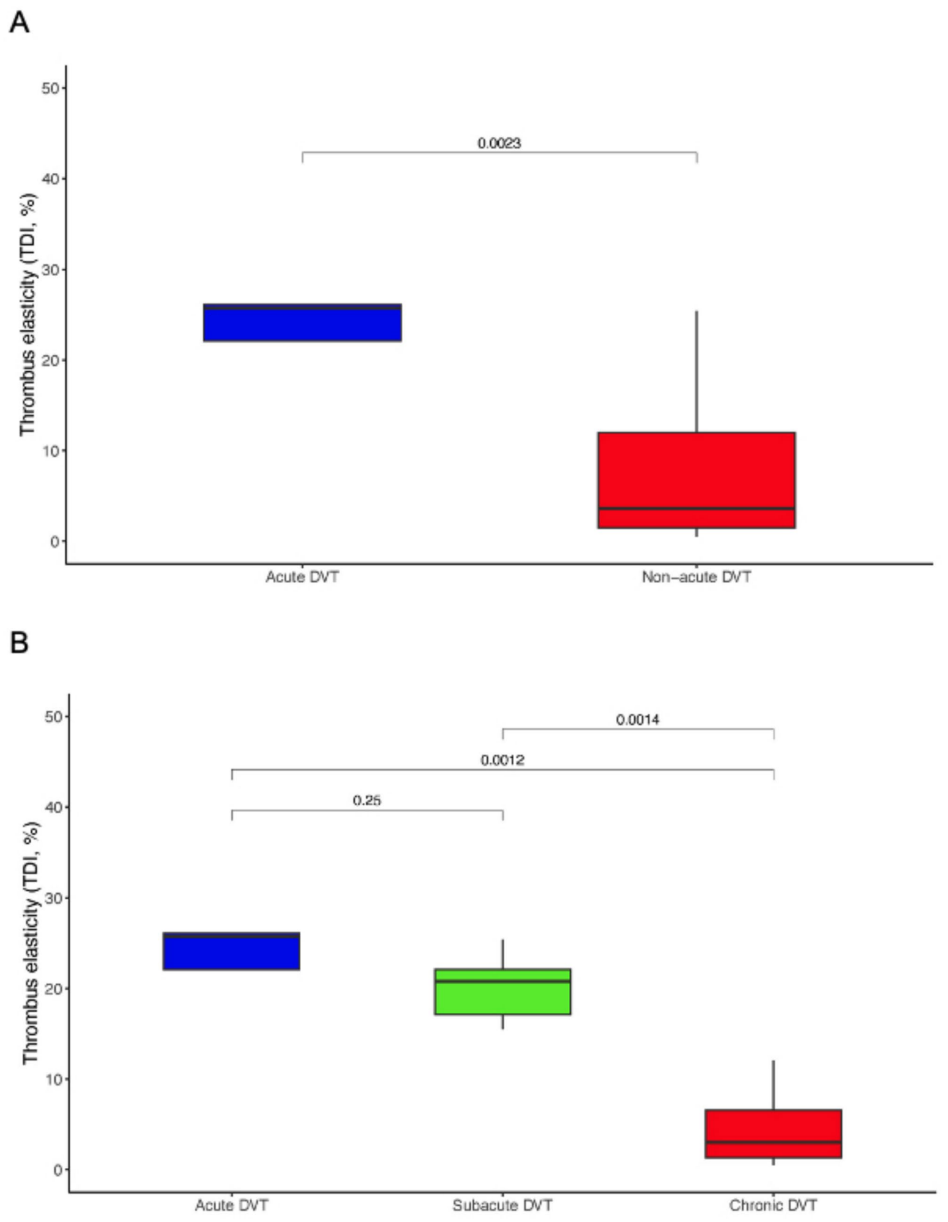
Thrombus elasticity according to DVT chronological age. Thrombus elasticity, assessed upon RT-SE using thrombus tissue deformation index, in **A** acute and non-acute DVT, and **B** acute, sub-acute, and chronic DVT

Based on the period of DVT diagnosis, five (13.2%) deep vein thrombi were categorized as acute. All patients in the acute DVT group had a D-dimer test performed which was positive based on age-adjusted thresholds, further potentially supporting the acuity of the thrombus. The remainder 33 (86.8%) thrombi were included in the non-acute DVT group. These were further categorized in to sub-acute (5, 13.2%) and chronic (28, 73.7%) thrombi. Median thrombus elasticity assessed by TDI was 25.7% (IQR, 22.1–26.1) in the acute DVT group, as opposed to 3.6% (IQR, 1.50–12.0) in the non-acute DVT group (*p* = 0.0023). Within the non-acute DVT group, sub-acute thrombi exhibited a median TDI value of 20.8% (IQR, 17.1–22.1) and chronic thrombi of 3.8% (IQR, 1.3–6.6; *p* < 0.001 for acute *vs* sub-acute *vs* chronic; Fig. 1).

Exclusion of distal thrombi (*n* = 5, all chronic) yielded consistent findings consistent with the main analysis reported above. When considering proximal DVTs only, the observed median TDI values for the non-acute and chronic proximal DVT subgroups were 5.2% (IQR, 1.8–15.9) and 3.5% (IQR, 1.6–8.3), respectively. Correspondingly, median thrombus elasticity in acute proximal DVT was approximately sevenfold greater that observed in chronic proximal DVT (25.7% [IQR, 22.1–26.1] vs 3.5% [IQR, 1.6–8.3]; *p* = 0.0018).

**Fig. 2:**
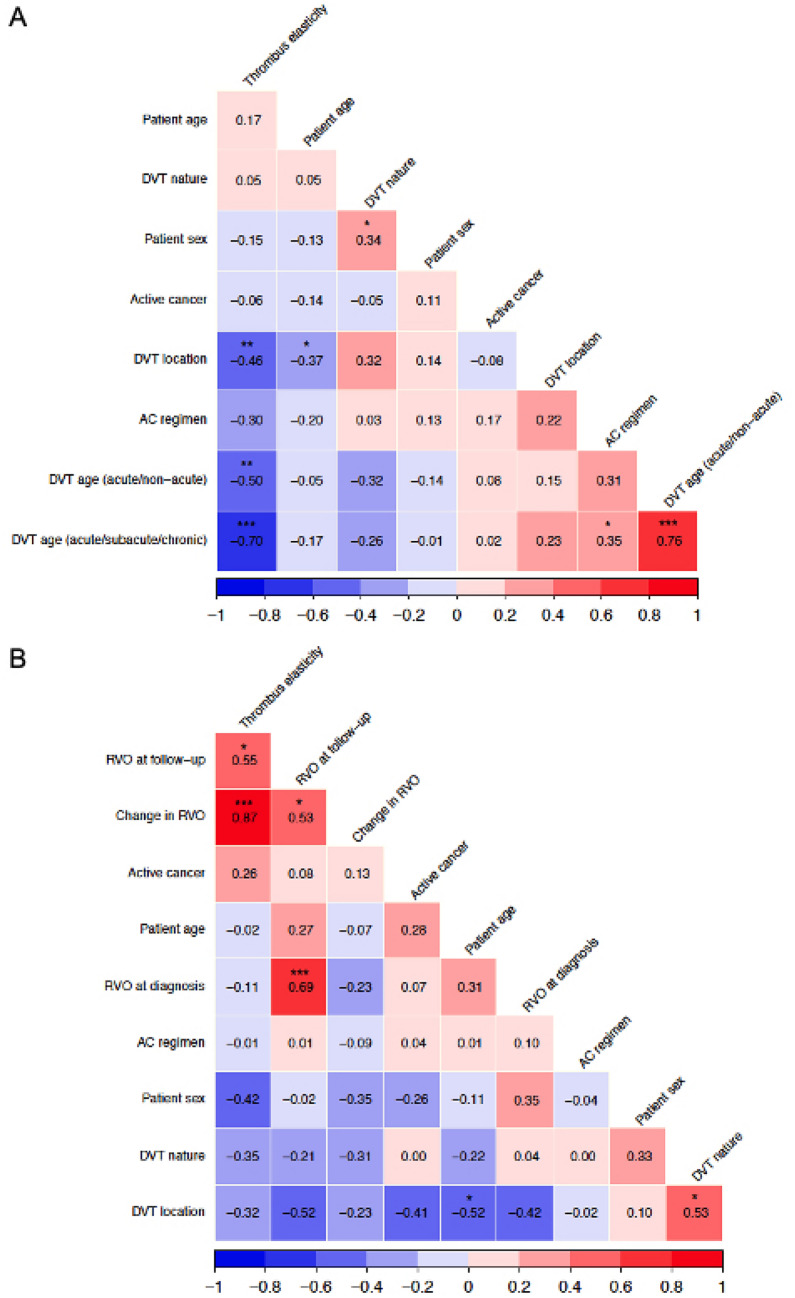
Correlations of thrombus elasticity with patient-, DVT-, and anticoagulation-related factors. Values in cells are Spearman’s rank-correlation coefficients (ρ [rho]). Asterisks indicate significance level (*p < 0.05; **p < 0.01; ***p < 0.001). Panel **A** shows results of correlation analysis considering all deep venous thrombi examined; panel **B** shows results of correlation analysis considering chronic deep venous thrombi only

In agreement with these observations, correlation analyses showed that thrombus elasticity was robustly associated with period of DVT diagnosis (*ρ* = − 0.50 for acute *vs* non-acute thrombi; *p* = 0.0012; and *ρ* = − 0.70 for acute *vs* sub-acute *vs* chronic thrombi; *p* < 0.0001), indicating lower thrombus elasticity (i.e., higher thrombus stiffness) with progressively increasing chronological age of DVT (Fig. 2). Thrombus elasticity also marginally correlated with DVT location (*ρ* = − 0.46; *p* = 0.0035), possibly suggesting a trend for overall lower elasticity in distal compared with proximal thrombi.

**Fig. 3:**
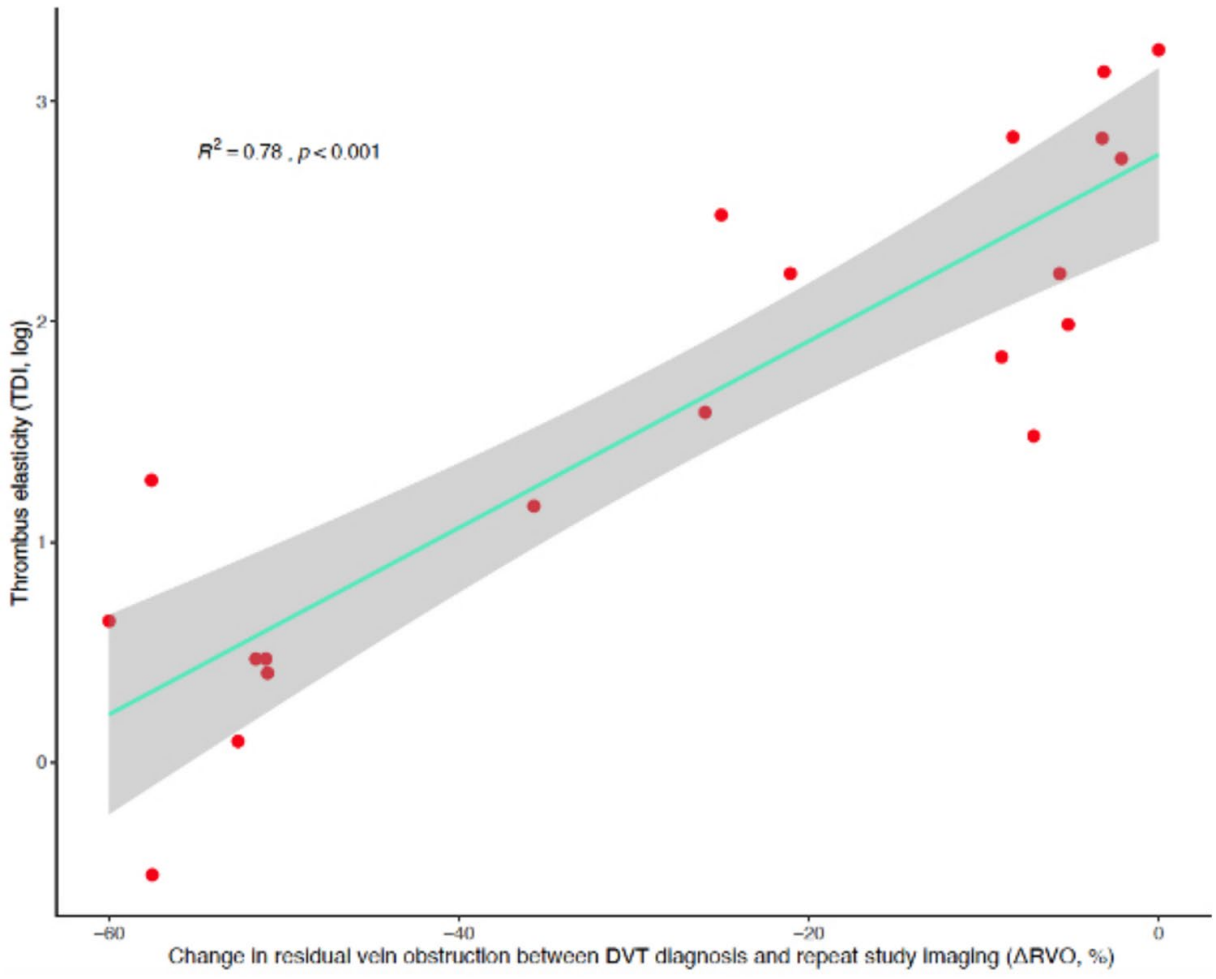
Thrombus elasticity in relation to DVT evolution

When restricting correlation analyses to chronic DVTs to explore associations of thrombus elasticity with DVT evolution over time, a robust relationship was detected between thrombus TDI and ΔRVO (*ρ* = 0.85; *p* < 0.0001), suggesting higher elasticity (i.e., lower stiffness) in thrombi undergoing smaller size reduction between diagnosis and repeat study imaging. A linear regression model shaping the relationship between the dynamic changes in RVO (independent variable) and the observed thrombus elasticity (dependent variable) exhibited good fitness overall (*R*^2^ = 78.0%; adjusted *R*^2^ = 76.4%; Fig. 3), predicting a mean 1.04% (95% confidence interval, 1.03–1.06; *p* < 0.0001) decrease in thrombus elasticity for every 1% reduction in ΔRVO.

## Discussion

In this prospective pilot study enrolling ambulatory patients with objectively confirmed lower-extremity DVT, we validated, for the first time to the best of our knowledge, the use of an advanced RT-SE-based software for quantitative thrombus elasticity assessment, and identified TDI as a novel promising imaging biomarker for assessment of thrombus-specific biomechanical features. For instance, thrombus elasticity estimated via TDI robustly correlated with the period of DVT diagnosis, with greater thrombus elasticity (i.e., higher TDI values) observed in acute DVT (< 14 days), and higher thrombus stiffness (i.e., lower TDI values) in chronic DVT (> 3 months). We found that the median thrombus elasticity, assessed using TDI, in acute DVT was approximately five- to seven-fold higher than that observed in non-acute DVT. Determining the chronological age of DVT can be of utmost importance across frequent clinical scenarios including a newly detected thrombus in individuals who are asymptomatic for DVT, or a suspected thrombus extension or recurrence in those with previous DVT [6]. In these settings, discriminating acute (or acutely recurring) DVT from stable chronic DVT is a major determinant in therapeutic decision-making, which may encompass initiation (or resumption/extension, in those with prior DVT) of anticoagulation in case the observed present clot is deemed to be acute, as opposed to no anticoagulation or treatment discontinuation in case the observed thrombus is considered to be chronic [6]. The biological rationale for correspondence between thrombus chronological age and its biomechanical properties is underpinned by multiple preclinical and ex vivo human studies, showing that acute thrombi tend to be more elastic/deformable as opposed to less elastic/deformable chronic thrombi [12–14]. DVT physiologically evolves over time. Acute thrombi primarily consist of loose fibrin mesh entrapping erythrocytes and platelets [15]. In sub-acute phases, cellular density increases due to immune cell infiltration regulating endogenous thrombolysis and inflammation [15]. In chronic phases, fibrosis pathways prevail, and thrombi undergo fibrous evolution with connective tissue deposition [15]. Elasticity imaging might, therefore, non-invasively provide clues into the complex intra-thrombus biology underlying DVT evolution and remodeling. [3, 7]

Previous studies suggested promise for RT-SE in dating DVT [7]. However, these employed the elastogram-generated color patterns to qualitatively label thrombus elasticity [16, 17], or arbitrarily derived empirical semi-quantitative parameters by comparing relative displacement of the thrombus tissue against that of a surrounding reference tissue [18, 19], which inherently introduce great amount of variability. Among these studies, *Li *et al*.* observed that approximately 70% of the examined clots were correctly classified as acute, sub-acute, or chronic based on to their elastogram color (i.e., strain map). In another study examining 26 acute (< 14 days) and 28 chronic (> 6 months) thrombi, *Rubin *et al*.* showed that relative strain, obtained by normalizing thrombus strain to that of the vein wall and the skin surface, was substantially higher in acute *vs* chronic DVT (2.75 *vs* 0.94, respectively; *p* < 0.0001), outperforming thrombus echogenicity in dating thrombosis diagnosis [18]. In another larger study by *Mumoli *et al*.*, semi-quantitative thrombus elasticity assessment, using a unitless scale representing relative strain values arbitrarily ranging from 0 (hardest) to 6 (softest), effectively discriminated acute from chronic proximal DVT with good diagnostic performance [19]. Whether RT-SE may help distinguish acute from sub-acute DVT has been poorly investigated to date and remains controversial. A previous study by *Yi *et al*.* suggested that RT-SE using strain ratio distinguished acute DVT from sub-acute DVT, which comprised thrombi diagnosed up to 6 months before [17]. In our study, while thrombus elasticity progressively decreased with increasing thrombus age, the difference in thrombus elasticity between acute and sub-acute DVT did not reach statistical significance. It should, however, be noted that, compared with the above-mentioned study [17], in the current study using a modern technology enabling quantitative elasticity assessment, a different and broadly used temporal classification of DVT acuity/chronicity was adopted, which may account for such discrepancy. In this regard, our findings are in agreement with another study by *Aslan *et al*.* that used a similar temporal DVT categorization, and did not detect major differences in elasticity patterns between acute and sub-acute DVT [16]. Our study supports a role for RT-SE in distinguishing acute or sub-acute DVT from chronic DVT, an aspect that may portend relevant implications with respect to anticoagulation decisions. Importantly, our study also provides a novel quantitative framework for thrombus elasticity assessment using modern RT-SE imaging enhanced with a commercially available, advanced quantitative software. Shear-wave elastography is another widely used elastography technique that employs acoustic radiation force impulses to generate shear waves within the tissue, being generally considered less operator-dependent and more reproducible by providing absolute stiffness estimates based on shear-wave velocity [4, 8]. Nevertheless, RT-SE has significantly broader availably, lower costs, and it can be added to the vast majority of the cardiovascular ultrasound scanner systems, making it suitable for wider scale clinical implementation including in vascular and thrombosis medicine practices [4, 8]. Studies directly comparing modern advanced RT-SE with shear-wave elastography in patients with DVT are lacking, and would be highly informative.

Our exploratory study also unveils previously unrecognized associations of thrombus elasticity with DVT evolution assessed by changes in RVO over time. Since RVO has been previously linked with an increased risk to develop the post-thrombotic syndrome (PTS) [20], our findings support future research evaluating thrombus elasticity as a potential predictor of adverse clinical outcomes after DVT including thrombus extension or embolization and PTS. In addition, it has been recently shown in preclinical venous thrombosis models and in thrombi derived from patients with venous thromboembolism or ischemic stroke that thrombus elasticity may affect clot responsiveness to thrombolysis or thrombectomy, potentially predicting embolization risk and treatment-related outcomes. [14, 21–23] As such, future studies exploring the role of thrombus elasticity imaging in assessing and predicting clot response to anticoagulation, thrombolysis, or other therapeutic interventions including anti-inflammatory therapies are warranted.

Our pilot study has several limitations, primarily being its small sample size. We enrolled patients with diverse clinical characteristics, including those with distal or cancer-associated DVT, where RT-SE use remained largely unexplored to date. It should, however, be noted that findings derived from the overall cohort might not uniformly apply to each individual subgroup. In particular, the use of ultrasound-based imaging may present inherent technical difficulties for smaller clots such as distal DVTs. To partly account for this, we repeated the main analysis excluding subjects with distal DVT, which did not alter the results. Whether thrombus elasticity is associated with other biologically and clinically relevant factors, (e.g., patient age or sex, circulating biomarkers, or different anticoagulation regimens) needs to be further investigated in larger studies. Importantly, RT-SE, especially when using non-quantitative approaches, is largely operator-dependent and subjected to intra-/inter-individual variability. We adopted multiple strategies to mitigate as much as possible these technical limitations which, however, cannot be definitely ruled out. The use of a modern RT-SE imaging system enhanced with an advanced quantitative elasticity analysis software further addressed, at least partly, some of these limitations. Lastly, reproducibility and generalizability of the current findings require adequate external validation. As such, these findings are to be be considered as hypothesis-generating.

## Conclusion

Our pilot study preliminary identified thrombus TDI as a potentially viable and clinically scalable elasticity imaging biomarker for assessment of thrombus biomechanical properties, potentially enabling enhanced non-invasive ultrasound-based characterization of lower extremity DVT. If further supported, modern RT-SE imaging implemented with advanced quantitative analysis software, might conveniently be combined with duplex ultrasonography (i.e., triplex ultrasonography) to provide adjunct thrombus-specific information, potentially advancing thrombus-guided management strategies in patients with DVT.

## Data Availability

Data are available upon reasonable request to the corresponding author.
